# Acute Kidney Injury Following Mini Percutaneous Nephrolithotomy for Renal Stones: predictors and Follow-up Evaluation in Real-life Setting

**DOI:** 10.1590/S1677-5538.IBJU.2025.0453

**Published:** 2025-09-30

**Authors:** Federica Passarelli, Ludovico Maria Basadonna, Fabio Ciamarra, Edoardo Bonacina, Giorgio Graps, Edoardo Sorba, Valentina Parolin, Gianpaolo Lucignani, Francesco Ripa, Stefano Paolo Zanetti, Fabrizio Longo, Elisa De Lorenzis, Giancarlo Albo, Emanuele Montanari, Luca Boeri

**Affiliations:** 1 IRCCS Fondazione Ca’ Granda Department of Urology Milan Italy Department of Urology, IRCCS Fondazione Ca’ Granda, Policlinico di Milano, Milan, Italy; 2 Dipartimento di Eccellenza Dipartimento di Scienze Cliniche e di Comunità Milan Italy Dipartimento di Scienze Cliniche e di Comunità, Dipartimento di Eccellenza, Milan, Italy

**Keywords:** Acute Kidney Injury, Nephrolithotomy, Percutaneous, Calculi

## Abstract

**Purpose:**

To evaluate the prevalence, predictors, and progression of acute kidney injury (AKI) in patients undergoing mPCNL for nephrolithiasis.

**Materials and methods:**

We retrospectively analyzed data from 569 patients who underwent mPCNL at a single tertiary academic center (01/2016-10/2024). AKI was defined per KDIGO criteria as sCr increase >0.3 mg/dL or ≥1.5× baseline. Stone-free status was no residual stones on CT at 3-month follow-up. Complications were classified using the modified Clavien system. Kidney function was reassessed 30–90 days post-op. Descriptive statistics, logistic regression, and Cox regression were applied.

**Results:**

Median (IQR) age and stone volume were 57 (48–66) years and 2.1 (0.9–4.2) cm³. Median preoperative sCr and operative time were 0.9 (0.7–1.1) mg/dL and 90 (73–120) minutes. Post-mPCNL, 40 patients (7.0%) developed AKI. Complications occurred in 138 (24.2%) patients; 449 (78.9%) were stone-free. AKI patients had higher CCI (1.3 vs. 0.5, p=0.04), pre-op sCr (1.1 vs. 0.8 mg/dL, p<0.01), stone volume (5.7 vs. 2 cm³, p=0.02), and longer operative time (130 vs. 90 min, p=0.01). Complications were more frequent in AKI patients (42.5% vs. 22.8%, p=0.01). At multivariate analysis, operative time (OR 1.1, p=0.03), pre-op sCr (OR 3.8, p=0.001), and early complications (OR 2.5, p=0.02) were independently associated with AKI. AKI persisted in 9 (22.5%) patients, mainly those with complications (88.9% vs. 38.7%, p=0.01). On Cox analysis, lower BMI (HR 0.8, p=0.02) and absence of complications (HR 0.3, p=0.01) predicted faster AKI recovery.

**Conclusion:**

Acute kidney injury remains a clinically significant complication following mPCNL.

## INTRODUCTION

Percutaneous nephrolithotomy (PCNL) is widely recognized as the gold standard surgical option for the management of large and anatomically complex renal stones, including staghorn and infection-related ones (1, 2). This procedure offers several advantages, such as high stone clearance rates, reduced surgical morbidity, and relatively rapid postoperative recovery, contributing to the widespread adoption of PCNL as the preferred approach for challenging renal stone disease (3, 4). Despite its clinical efficacy, PCNL is associated with various postoperative complications, ranging from minor issues, such as fever and transient urinary leakage, to more serious outcomes, like significant hemorrhage, sepsis, and other infection-related morbidities (5). These complications can significantly impact not only immediate postoperative recovery but also long-term renal function and overall patient prognosis. Reported rates of overall complications following PCNL vary considerably, ranging from 2% to 17.1%, (5) which underscores the importance of thorough perioperative assessment and individualized postoperative monitoring to mitigate risks and improve clinical outcomes. Among the recognized postoperative complications, AKI has recently emerged as an important clinical concern. AKI is characterized by a sudden and sustained decline in renal function, which can lead to impaired postoperative recovery and an increased risk of further morbidity and adverse clinical outcomes (6). The pathophysiology of AKI in the context of PCNL is complex and multifactorial, encompassing surgical trauma, ischemic injury, infection, and exposure to nephrotoxic agents (7). Despite its clinical significance, the incidence, natural history, and long-term impact of AKI occurring in the early postoperative period following PCNL remain poorly defined. Existing literature on the topic is limited, with studies reporting highly variable incidence rates of postoperative AKI ranging from 4% to 24% (8-10). This variability likely reflects differences in patient characteristics, surgical techniques, and perioperative management protocols. Of note, all previous studies have primarily focused on standard PCNL, whereas the prevalence and predictors of AKI following mPCNL have not been specifically investigated. Although mPCNL is generally associated with a lower rate of overall complications compared to standard PCNL, (1, 4, 11, 12) the impact of this technique on postoperative renal function, and particularly on AKI, remains unexplored in the current literature. Thereof, our study aimed to evaluate the prevalence of and to identify clinical and procedural predictors of AKI in a real-life cohort of patients undergoing mPCNL for nephrolithiasis. Additionally, we sought to explore the temporal evolution of post-procedural renal function, assessing both early postoperative period and longer-term outcomes, with a specific focus on persistence of AKI over time. In doing so, we sought to provide valuable insights that can enhance risk stratification, guide perioperative management, and ultimately improve the safety and efficacy of mPCNL in clinical practice.

## MATERIALS AND METHODS

We retrospectively analyzed data from 637 patients who underwent mPCNL for kidney stones at a tertiary referral academic center between January 2016 and October 2024. Demographic, clinical and laboratory data were collected. Body weight and height were recorded to calculate each participant's body mass index (BMI). Health-relevant comorbidities were assessed using the Charlson Comorbidity Index (CCI) (13). Stone characteristics, including mean stone density in Hounsfield units (HU), presence of multiple and staghorn stones, were assessed in all patients prior to surgery via non-contrast-enhanced computed tomography (CT). Stone volume was calculated using the ellipsoid formula (length × width × height × π×1/6) (14).

All surgical procedures were conducted under general anesthesia by three high-volume, experienced endourologists. Following ureteral catheter placement, mPCNL was carried out using either the 16 F ClearPetra set (vacuum-assisted mPCNL, vamPCNL) (15, 16) or the MIP 16 F metallic sheath (vacuum-cleaner mPCNL, vcmPCNL) (4) in combination with a 12 F MIP nephroscope and a Holmium laser (VersaPulse PowerSuite 100 W; Lumenis, Israel). Stone fragmentation was performed using a 550-µm holmium: YAG laser fiber set to short-pulse mode, with energy parameters ranging from 1.2 to 1.5 joules and a frequency of 20 to 30 Hz, adjusted according to intraoperative needs. An 8 F nephrostomy tube was used as the only drainage method, and the ureteral catheter was removed immediately post-surgery. During the procedure, contrast medium was administered exclusively within the urinary tract. Pain management included fixed-dose acetaminophen on the first postoperative day, along with nonsteroidal anti-inflammatory drugs (NSAIDs) administered if needed. Starting from the second postoperative day, NSAIDs were discontinued, and acetaminophen was given on an as-needed basis, depending on the patient's individual pain requirements. As for antibiotic prophylaxis, a single dose of antibiotic was administered preoperatively. The use of multiple percutaneous access tracts was recorded. Operative time was measured from the initial puncture to the completion of stone removal.

Postoperative outcomes included hospitalization time, postoperative serum creatinine and hemoglobin, and overall complication rate, which was classified according to the PCNL-adjusted Clavien-Dindo classification (17, 18).

According to The Kidney Disease: Improving Global Outcome definition and staging system (KDIGO) criteria AKI was defined as an increase in serum creatinine (SCr) of >0.3 mg/dL within 48 h or to ≥ 1.5 times the preoperative baseline.(6) Kidney function was re-evaluated 30 to 90 days after mPCNL to identify persistent AKI. Patients underwent non contrast-enhanced CT scan at three months postoperatively to assess the presence of residual fragments (19, 20).

We excluded patients with preoperative renal impairment (estimated glomerular filtration rate <60 mL/min/1.73 m^2^; n = 28), those with indwelling ureteral stents or nephrostomy tubes (n = 14), patients scheduled for staged procedures (n = 29), and those with symptomatic urinary tract infections or who had undergone other kidney surgeries within 6 months prior to mPCNL (n = 18). A final cohort of 569 patients who underwent mPNL for kidney stones was available for statistical analysis.

Data collection followed the principles outlined in the Declaration of Helsinki. All patients signed an informed consent agreeing to share their own anonymous information for future studies. The study was approved by our Hospital Ethical Committee (Prot. 25508).

## Statistical analysis

The sample size was calculated as follows. Based on previous studies reporting a 16% incidence of postoperative AKI in patients undergoing PCNL,(18) and assuming a 95% confidence level with a Type I error rate (α) of 0.05, the minimum required sample size was estimated to be 207 patients (Russ-Lenth applet for Windows).

Distribution of data was tested with the Shapiro–Wilk test. Data are presented as medians (interquartile range; IQR) or frequencies (proportions). Descriptive statistics were used to characterize the entire study cohort. Subsequently, differences in clinical and operative parameters between patients who developed postoperative AKI and those who did not were assessed using the Mann–Whitney U test and Fisher Exact test, as appropriate. Univariate and multivariate logistic regression analyses were performed to identify potential predictors of AKI following mPCNL. Lastly, Cox regression model was employed to identify factors associated with the recovery of kidney function in the subgroup of patients who developed AKI.

Statistical analyses were performed using SPSS v.28 (IBM Corp., Armonk, NY, USA). All tests were two sided and statistical significance level was set at p<0.05.

## RESULTS

Table-1 depicts demographic and clinical characteristics of the whole cohort. Median patients’ age was 57 (48–66) years, and 229 (40.2%) patients were female. Median BMI was 24.6 (22.0–27.7) kg/m^2^ and 214 (37.6%) participants had a CCI ≥1. At baseline, median serum creatinine and estimated Glomerular Filtration Rate (eGFR) were 0.9 (0.7–1.1) mg/dL and 84.6 (66.6-97.5) mL/min/1.73 m^2^, respectively. Median stone volume was 2.1 (0.9–4.2) cm³, with multiple stones observed in 349 (61.3%) patients. Above all, 487 (85.6%) patients underwent vamPCNL, while 82 (14.4%) underwent vcmPCNL, with a median operative time of 90 (73–120) minutes (Table-1).

**Table 1:** Demographic characteristics of the study cohort (N = 569).

Age (years)		
	Median (IQR)	57 (48-66)
	Range	19-85
**Female Gender [No. (%)]**		229 (40.2)
**BMI (kg/m^2^)**		
	Median (IQR)	24.6 (22.0-27.7)
	Range	17.9 – 40.1
**CCI**		
	Mean (SD)	0.7 (1.1)
	Median (IQR)	0 (0-1)
	Range	0–8
CCI ≥1 [No. (%)]		214 (37.6)
**Baseline serum creatinine (mg/dL)**		
	Median (IQR)	0.9 (0.7-1.1)
	Range	0.4–1.5
**Baseline eGFR (mL/min/1.73 m^2^)**		
	Median (IQR)	84.6 (66.6-97.5)
	Range	60.1–100.4
**Baseline haemoglobin (g/dL)**		
	Median (IQR)	13.9 (12.2-15.1)
	Range	10.4 – 16.9
Hypertension [No. (%)]		163 (28.6)
**Stone volume (cm^3^)**		
	Median (IQR)	2.1 (0.9-4.2)
	Range	0.5 – 53.1
Multiple stones [No. (%)]		349 (61.3)
Staghorn stone [No. (%)]		143 (25.1)
**Mean stone density (Hounsfield unit)**		
	Median (IQR)	834 (585-1031)
	Range	150–1983
**mPCNL procedure type [No. (%)]**		
	vamPCNL	487 (85.6)
	vcmPCNL	82 (14.4)
Multiple access tracts [No. (%)]		78 (13.7)
**Operative time (min.)**		
	Median (IQR)	90 (73-120)
	Range	30 – 180
**Postoperative serum creatinine (mg/dL)**		
	Median (IQR)	0.9 (0.8-1.2)
	Range	0.4 – 5.5
**Postoperative haemoglobin (g/dL)**		
	Median (IQR)	12.5 (11.2-13.7)
	Range	7.5 – 15.2
**Hospitalization time (days)**		
	Median (IQR)	4 (3-6)
	Range	2 – 50
Acute kidney injury [No. (%)]		40 (7.0)
Any complications [No. (%)]		138 (24.2)
**Postoperative complications [No. (%)]**		
(Highest Clavien score)		
	Clavien-Dindo I-II	118 (20.7)
	Clavien-Dindo IIIa/b	20 (3.5)
	Stone free rate [No. (%)]	449 (78.9)

Keys: BMI = body mass index; CCI = Charlson Comorbidity Index; eGFR = estimated Glomerular Filtration Rate; vamPCNL = Vacuum-assisted mini-percutaneous nephrolithotomy; vcmPCNL = Vacuum-cleaner mini-percutaneous nephrolithotomy.

Postoperatively, AKI occurred in 40 (7.0%) patients, while overall postoperative complications were observed in 138 (24.2%) participants. According to the Clavien-Dindo classification, 118 (20.7%) patients experienced grade I–II complications, and 20 (3.5%) had grade IIIa or IIIb complications. Stone-free rate was 78.9% following a single mPCNL procedure (Table-1).

Table-2 depicts demographic and clinical characteristics of the whole cohort according to the presence of postoperative AKI. Patients with AKI had higher CCI (p=0.04) and worse baseline renal function (p<0.01) than those without AKI. Stone volume was significantly larger in AKI patients (5.7 vs. 2.0 cm³; p=0.02) and AKI was more frequently found among those with multiple (77.5% vs. 60.1%; p=0.03) and staghorn stones (47.5% vs. 23.4%; p=0.01). Operative time was also significantly longer in patients with AKI (130 vs. 90 minutes; p=0.01). Overall, patients with AKI more frequently had post-operative complication (42.5% vs. 22.8%; p=0.01) than those without AKI.

**Table 2:** Demographic characteristics of the study cohort according to postoperative AKI (N = 569).

		AKI	+ AKI	p value*
No. of patients [No. (%)]		529 (93.0)	40 (7.0)	
**Age (years)**				0.2
	Median (IQR)	55 (47-62)	55 (51-68)	
	Range	19–85	23-79	
Female Gender [No. (%)]		214 (40.5)	15 (37.5)	0.2
**BMI (kg/m^2^)**				0.8
	Median (IQR)	24.4 (21.6-27.4)	27.3 (20.5-28.8)	
	Range	17.9–38.5	18.1–40.1	
**CCI**				0.04
	Mean (SD)	0.5 (0.9)	1.3 (1.5)	
	Median (IQR)	0 (0-1)	1 (0-2)	
	Range	0–6	0-8	
CCI ≥1 [No. (%)]		193 (36.4)	21 (52.5)	0.03
	Baseline serum creatinine (mg/dL)			<0.01
	Median (IQR)	0.8 (0.7-1.1)	1.1 (0.9-1.4)	
	Range	0.4–1.5	0.5–1.5	
**Baseline eGFR (mL/min.)**				<0.01
	Median (IQR)	89.6 (68.7-90.5)	72.5 (60.2-85.3)	
	Range	60.4–100.4	60.1–90.5	
**Baseline haemoglobin (g/dL)**				0.8
	Median (IQR)	14.0 (12.1-15.0)	14.2 (12.1-15.3)	
	Range	11.4–16.9	10.4–14.5	
Hypertension [No. (%)]		133 (25.1)	12 (30.0)	0.4
**Stone volume (cm^3^)**				0.02
	Median (IQR)	2.0 (0.8-4.1)	5.7 (1.8-11.4)	
	Range	0.5–43.2	1.0–53.1	
Multiple stones [No. (%)]		318 (60.1)	31 (77.5)	0.03
Staghorn stone [No. (%)]		124 (23.4)	19 (47.5)	0.01
**Mean stone density (Hounsfield unit)**				0.6
	Median (IQR)	860 (614-1042)	940 (679-1187)	
	Range	150–1983	372-1563	
**mPCNL procedure type [No. (%)]**				0.1
	vamPCNL	450 (85.1)	37 (92.5)	
	vcmPCNL	79 (14.9)	3 (7.5)	
Multiple access tracts [No. (%)]		72 (13.6)	6 (15.0)	0.8
**Operative time (min.)**				0.01
	Median (IQR)	90 (75-128)	130 (98-161)	
	Range	30–160	56-180	
**Postoperative serum creatinine (mg/dL)**				<0.01
	Median (IQR)	0.9 (0.7-1.1)	1.7 (1.3-2.1)	
	Range	0.4–2.2	0.7–5.5	
**Postoperative haemoglobin (g/dL)**				0.1
	Median (IQR)	12.3 (11.1-13.6)	12.1 (10.1-13.0)	
	Range	7.5–15.2	9.6–14.9	
**Hospitalization time (days)**				0.03
	Median (IQR)	4 (3-6)	5 (3-9)	
	Range	2–20	3-50	
Any complications [No. (%)]		121 (22.8)	17 (42.5)	0.01
**Postoperative complications [No. (%)]**				0.3
(Highest Clavien score)				
	Clavien-Dindo I-II	108 (20.4)	10 (25.0)	
	Clavien-Dindo IIIa/b	19 (3.6)	1 (2.5)	
Stone free rate [No. (%)]		422 (79.7)	27 (67.5)	0.1

Keys: AKI = acute kidney injury; BMI = body mass index; CCI = Charlson Comorbidity Index;

vamPCNL = Vacuum-assisted mini-percutaneous nephrolithotomy;

vcmPCNL = Vacuum-cleaner mini-percutaneous nephrolithotomy;

* P value according to the Mann-Whitney U test for continuous data and the Fisher Exact Test for categorical variables, as indicated

Table-3 depicts univariate (UVA) and multivariate (MVA) logistic regression models testing the associations between clinical variables and post-operative AKI. At MVA, higher baseline serum creatinine (OR 3.8, p=0.001), longer operative time (OR 1.1, p=0.03) and the occurrence of postoperative complications (OR 2.5, p=0.02) were independently associated with an increased risk of postoperative AKI, after accounting for CCI.

**Table 3:** Logistic regression models predicting acute kidney injury after surgery in the whole cohort.

UVA model		MVA model		
	**OR, p-value**	**95% CI**	**OR, p-value**	**95% CI**
Age	1.1, 0.3	0.98–1.13		
Female gender	0.9, 0.8	0.49-1.19		
CCI	1.4, 0.01	1.09-1.87	1.2; 0.2	0.94–1.45
Baseline serum creatinine	3.7; 0.001	1.72–7.83	3.8; 0.001	1.82–7.95
Stone Volume	1.1; 0.04	1.04-1.39		
Stone density (HU)		1.0; 0.6	0.98-1.03	
Operative time	1.1; 0.02	1.02-1.44	1.1; 0.03	1.03–1.75
vamPCNL vs vcmPCNL	1.2; 0.2	0.60–5.62		
Complications	2.4; 0.01	1.13–5.04	2.5; 0.02	1.15–5.33
Stone free status	0.7; 0.4	0.27–1.73		

Keys: UVA = Univariate model; MVA = Multivariate model, CCI = Charlson Comorbidity Index;

vamPCNL = Vacuum-assisted mini-percutaneous nephrolithotomy;

vcmPCNL = Vacuum-cleaner mini-percutaneous nephrolithotomy;

Table-4 presents demographic and clinical characteristics of the study cohort according to persistence of AKI. Out of 40 patients with AKI after mPCNL, 9 (22.5%) had persistent AKI at a median follow-up of 60 (45-100) days. There were no significant differences between the two groups in terms of baseline demographics, comorbidities, stone characteristics, or operative parameters. A significantly higher prevalence of hypertension was observed in patients with persistent AKI compared to those without (55.6% vs. 22.6%, p=0.04). Moreover, patients with persistent AKI more frequently reported post mPCNL complications (88.9% vs. 38.7%, p=0.01), and were more likely to suffer higher-grade postoperative complications (44.4% vs. 3.2%, p=0.03). Finally, patients with residual stone fragments after mPCNL more frequently showed persistent AKI compared to those stone free (p=0.02).

**Table 4:** Demographic characteristics of the study cohort according to persistent AKI (N = 40).

		No persistent AKI	Persistent AKI	p value*
**No. of patients [No. (%)]**		**31 (77.5)**	**9 (22.5)**	
**Age (years)**				0.5
	Median (IQR)	61 (52-65)	59 (53-69)	
	Range	46–70	53-73	
Female Gender [No. (%)]		10 (32.2)	5 (55.5)	0.2
**BMI (kg/m** ^2^ **)**				0.7
	Median (IQR)	27.1 (21.2-28.8)	31.9 (27.5-32.1)	
	Range	17.9–30.8	27.7–40.1	
**CCI**				0.3
	Mean (SD)	1.1 (1.2)	1.3 (1.5)	
	Median (IQR)	1 (0-2)	2 (1-2)	
	Range	0–4	1-4	
**Baseline serum creatinine (mg/dL)**				0.2
	Median (IQR)	1.1 (0.8-1.8)	1.2 (0.9-1.9)	
	Range	0.4-1.5	0.9–1.5	
**Baseline haemoglobin (g/dL)**				0.9
	Median (IQR)	13.4 (11.7-14.8)	13.3 (12.6-15.2)	
	Range	10.5–13.6	12.6–15.8	
Hypertension [No. (%)]		7 (22.6)	5 (55.6)	0.04
**Stone volume (cm3)**				0.8
	Median (IQR)	4.4 (1.4-15.3)	5.4 (2.9-19.1)	
	Range	0.5–32.6	1.1–53.0	
Multiple stones [No. (%)]		22 (71.0)	6 (75.0)	0.8
Staghorn stone [No. (%)]		16 (51.6)	4 (44.4)	0.7
**Mean stone density (Hounsfield unit)**				0.3
Median (IQR)		1031 (692-1241)	980 (686-1220)	
Range		370–1574	466-1023	
vamPCNL procedure type [No. (%)]		26 (83.9)	8 (88.9)	0.7
Multiple access tracts [No. (%)]		6 (19.4)	2 (22.2)	0.9
**Operative time (min)**				0.5
Median (IQR)		115 (97-160)	135 (110-154)	
	Range	56–110	110-180	
**Postoperative serum creatinine (mg/dL)**				0.4
	Median (IQR)	1.6 (0.7-1.1)	1.8 (1.3-2.1)	
	Range	0.7–5.8	1.6–2.1	
**Postoperative haemoglobin (g/dL)**				0.4
	Median (IQR)	11.7 (9.7-12.9)	9.8 (10.1-13.0)	
	Range	7.5–15.6	8.1–12.6	
**Hospitalization time (days)**				0.2
	Median (IQR)	5 (3-10)	6 (4-12)	
	Range	3–50	3-42	
Any complications [No. (%)]		12 (38.7)	8 (88.9)	0.01
**Postoperative complications [No. (%)]**				0.03
(Highest Clavien score)				
	Clavien-Dindo I-II	11 (35.5)	4 (44.4)	
	Clavien-Dindo IIIa/b	1 (3.2)	4 (44.4)	
	Stone free rate [No. (%)]	23 (74.2)	3 (33.3)	0.02

Keys: AKI = acute kidney injury; BMI = body mass index; CCI = Charlson Comorbidity Index;

vamPCNL = Vacuum-assisted mini-percutaneous nephrolithotomy;

vcmPCNL = Vacuum-cleaner mini-percutaneous nephrolithotomy;

* P value according to the Mann-Whitney U test for continuous data and the Fisher Exact Test for categorical variables, as indicated

Table-5 reports Cox regression analysis evaluating predictors of AKI recovery in the entire cohort. Higher BMI (HR 0.8, p=0.02) and the presence of postoperative complications (HR 0.3, p=0.01) were associated with a delayed recovery from AKI. No other variables were significantly associated with AKI recovery.

**Table 5:** Cox regression model of AKI recovery (HR; p value [95% CI]) in the whole cohort.

	HR	p-value	95% CI
Age	0.9	0.3	0.95–1.02
BMI	0.8	0.02	0.76–0.97
Female gender	0.9	0.9	0.41-2.20
CCI	1.1	0.1	0.93-1.76
Baseline serum creatinine	0.8	0.9	0.51–2.17
Stone Volume	1.1	0.2	0.95-1.17
Stone density	1.1	0.1	0.93-1.03
Operative time	0.9	0.7	0.98-1.12
vamPCNL vs vcmPCNL	1.3	0.5	0.51–4.05
Complications	0.3	0.01	0.16–0.74
Stone free status	1.0	0.4	0.74–1.84

Keys: BMI = Body Mass Index; CCI = Charlson Comorbidity Index;

vamPCNL = Vacuum-assisted mini-percutaneous nephrolithotomy;

vcmPCNL = Vacuum-cleaner mini-percutaneous nephrolithotomy;

## DISCUSSION

The primary aim of our study was to assess the prevalence of AKI in a real-life cohort of patients undergoing mPCNL for kidney stones, and to identify demographic, clinical, and procedural factors independently associated with its development. Moreover, we investigated predictors of persistent renal function impairment at a median follow-up of 60 (45-100) days. This comprehensive approach provides novel insights into both the immediate renal impact of mPCNL, and the risk factors associated with ongoing renal dysfunction, with the goal of informing perioperative risk stratification and improving renal outcomes in this patient population.

We showed that, in our cohort, postoperative AKI occurred in 7% of cases and was associated with distinct clinical and procedural characteristics. Patients who developed AKI had worse baseline renal function, longer operative times and more frequently experienced postoperative complications than those without AKI. While most cases of AKI were transient, one out of five participants had persistent renal dysfunction at follow-up. Persistence of AKI was associated with hypertension, higher-grade postoperative complications, and a lower likelihood of achieving stone-free status. Furthermore, higher BMI and the presence of complications were found to be associated with a slower recovery from AKI at follow-up.

To the best of our knowledge, this is the first study specifically investigating the incidence, risk factors, and persistence of AKI in patients undergoing mPCNL. Prior studies have evaluated AKI following standard PCNL (7), which typically employs larger tract sizes and may be associated with greater renal parenchymal trauma. In contrast, mPCNL is considered a less invasive alternative, associated with reduced postoperative complications and lower risk of renal injury (11). The incidence of AKI in our study, at 7%, is comparable to the range reported in the literature, which varies between 4% and 24% (7, 9, 10). Despite the smaller tract size and lower morbidity, our findings demonstrate that AKI remains a clinically significant complication even in the context of mPCNL. This observation underscores that while miniaturized techniques may offer advantages in terms of bleeding, infection and postoperative pain (21,22), they do not preclude the risk of acute postoperative renal impairment.

Several factors have been previously identified as contributors to AKI following PCNL, including impaired baseline renal function, prolonged operative time, and perioperative complications such as bleeding or infection (7). Our findings are consistent with this evidence, confirming the role of both patient-related and procedural variables in influencing renal outcomes. In particular, the association between worse baseline renal function and AKI aligns with prior studies demonstrating that reduced renal reserve predisposes to further injury under surgical stress (7). Longer operative times have similarly been implicated as an independent risk factor, possibly due to increased intrarenal pressure, thermal injury, or cumulative parenchymal manipulation. Importantly, this association extends beyond urologic procedures, indeed studies in cardiac surgery have demonstrated that prolonged surgical times are significant predictors of postoperative AKI (23). Furthermore, postoperative complications, frequently linked to systemic inflammation or hemodynamic instability, have been repeatedly shown to contribute to AKI pathophysiology, as also shown in a review by Ostermann et al. (24). In this context, reducing intrarenal pressure and operative time is crucial to minimize the risk of post-PCNL complications (16, 22, 25). Indeed, our results showed that postoperative complications were independently associated with the risk of AKI and with a delayed recovery from AKI at follow-up.

The literature corroborates the detrimental impact of postoperative complications on renal outcomes. Indeed, as shown by Mishra et al., infectious and hemorrhagic events have a well-documented detrimental effect on renal outcomes by promoting systemic inflammation and hemodynamic instability, thereby increasing the risk of sustained AKI (26). Additionally, presence of residual fragments not only predisposes to infection and inflammation but has been linked to poorer recovery trajectories in renal function (19).

Also, while our MVA was structured to assess predictors of AKI rather than complications, our data suggest that AKI may not only be a consequence of perioperative complexity and postoperative complication occurrence, but also a marker of patient vulnerability and procedural burden, potentially contributing to worse postoperative courses. This interpretation aligns with prior evidence linking AKI to increased morbidity and supports the notion that AKI itself could serve as a clinically relevant early indicator of adverse outcomes after mini-PCNL (24).

Therefore, miniaturized and vacuum-assisted procedures, which have been associated with higher SFR and lower complication rates compared to standard ones, should be preferred to minimize the impact of PCNL on immediate and short-term renal function (19, 20, 25).

Furthermore, higher BMI, which we found to be an independent predictor of slower renal recovery, has also been recognized in other surgical contexts as a risk factor for AKI development and delayed renal healing. Notably, a retrospective study of critically ill patients showed that obesity doubled the risk of AKI even after adjusting for illness severity (27).

Similarly, according to our findings, pre-existing hypertension has been independently associated with increased susceptibility to AKI, likely due to chronic vascular and renal structural changes that reduce renal reserve and impair adaptive responses to surgical stress (8).

In conclusion, our study provides novel insight into the incidence and predictors of postoperative AKI in patients undergoing mPCNL. Our findings confirm the prognostic value of baseline serum creatinine, which emerged as the strongest independent predictor of postoperative AKI after mini-PCNL, suggesting that even minor variations in baseline renal reserve may significantly influence postoperative renal outcomes. Despite the minimally invasive nature of mPCNL, AKI occurred in 7% of patients and was significantly associated with baseline renal dysfunction, prolonged operative time, and the occurrence of postoperative complications, highlighting the role of procedural complexity and perioperative physiological stress in renal injury. Moreover, a subset of patients experienced persistent renal impairment, with higher BMI, presence of residual fragments and postoperative complications emerging as relevant predictors. Given that all procedures were performed by experienced urologists using standardized mPCNL techniques, these associations likely reflect intrinsic patient and stone-related factors rather than technical variability. These results underscore the importance of preoperative risk stratification based on serum creatinine and suggest that technical refinements aimed at minimizing operative time and reducing intraoperative stress may play a role in preventing renal injury. Therefore, optimization of renal function before surgery, meticulous intraoperative stone clearance, and implementation of strategies to reduce postoperative complications may be critical for optimizing renal outcomes. Further prospective, multicentric studies with long-term follow-up are warranted to validate our findings and refine strategies to mitigate AKI risk in this population and further personalize surgical management.

Despite being innovative, our study is not devoid of limitations. First, its retrospective, single-center design may limit the generalizability of the findings. Second, renal function was assessed solely through serum creatinine, which may not fully capture subtle or subclinical changes in renal performance and can be influenced by non-renal factors. Additionally, direct measure of intraoperative renal pressure was not performed. To minimize confounding, we excluded patients with preoperative renal impairment, indwelling stents or nephrostomy tubes, staged procedures, symptomatic UTIs, and recent renal surgery; however, this may have led to an underestimation of AKI incidence in a broader clinical population. Lastly, the follow-up duration was limited, and long-term renal outcomes beyond the early postoperative period were not systematically evaluated, precluding conclusions on the chronic implications of post-mPCNL AKI.

## CONCLUSIONS

This study demonstrates that acute kidney injury remains a clinically significant complication following mPCNL. Independent predictors of AKI and its persistence include worse baseline renal function, prolonged operative time, higher BMI and postoperative complications. These findings underscore the importance of comprehensive perioperative risk assessment and optimization strategies. Prospective, multicenter investigations with extended follow-up are warranted to validate these observations and guide clinical practice.

## Data Availability

All data generated or analysed during this study are included in this published article
